# Quality of whole genome sequencing from blood versus saliva derived DNA in cardiac patients

**DOI:** 10.1186/s12920-020-0664-7

**Published:** 2020-01-29

**Authors:** Roderick A. Yao, Oyediran Akinrinade, Marie Chaix, Seema Mital

**Affiliations:** 1 Genetics and Genome Biology, Hospital for Sick Children, 555 University Ave, Toronto, Ontario M5G 1X8 Canada; 2 Department of Pediatrics, Hospital for Sick Children, 555 University Ave, Toronto, Ontario M5G 1X8 Canada; 3 University of Toronto, 27 King’s College Circle, Toronto, Canada

**Keywords:** Human genome, Genomics, DNA, Whole genome sequencing, Single nucleotide variants, Saliva, Cardiovascular disease

## Abstract

**Background:**

Whole-genome sequencing (WGS) is becoming an increasingly important tool for detecting genomic variation. Blood derived DNA is the current standard for WGS for research or clinical purposes but may not always be feasible to acquire. The usability of DNA from saliva for WGS is not known. We compared the quality of WGS between blood versus saliva derived DNA.

**Methods:**

WGS was performed in DNA from 531 blood and 502 saliva samples (including 5 paired samples) from participants enrolled in a heart disease biorepository. We compared the proportion of sequencing reads that mapped to non-human sources (microbiome), the sequencing coverage, and the yield and concordance of single nucleotide variant (SNV) and copy number variant (CNV) calls between blood and saliva genomes.

**Results:**

Of 531 blood and 502 saliva samples, 46% saliva DNA failed quality control (QC) requirements for WGS compared to 6% QC failure for blood DNA. An average of 10.7% WGS reads in the saliva samples mapped to the human oral microbiome compared to 0.09% WGS reads in blood samples. However, these reads were readily excluded by excluding reads that did not map to the human reference genome. Sequencing coverage met or exceeded the target sequencing depth of 30x in all the blood samples and 4 of the 5 saliva samples; the fifth saliva sample had an average sequencing depth of 22.6x. Over 95% of SNVs identified in saliva were concordant with those identified in blood across the genome, within all gene coding regions, and within cardiovascular disease-related gene coding regions. Rare SNVs, defined as those with a minor allele frequency of less than 1% in the Genome Aggregation Database, had a lower concordance of 90% between blood and saliva genomes. CNVs had only 76% concordance between blood and saliva samples.

**Conclusions:**

High quality saliva samples that meet stringent QC criteria can be used for WGS when blood-derived DNA is not available or is not suitable. Saliva DNA provides an acceptable yield of SNV calls but has a lower yield for CNV calls compared to blood DNA.

## Background

As the cost of sequencing continues to decrease, whole genome sequencing (WGS) is being increasingly used for both research and clinical applications to study the role of genetic variation in human disease. The use of blood derived DNA is the current standard for WGS. Published studies report that saliva-derived DNA can be used for array genotyping [1] and whole-exome sequencing [2] as long as the quantity of human DNA in each sample is sufficient. Wall et al. [3] reported finding no differences in sequencing quality or variant call error rate between blood and saliva samples for both whole exome sequencing (WES) and WGS. However, there have been very few studies comparing WGS results from paired blood and saliva-derived DNA.

The advantages of using saliva for sequencing are the ability to get a sample non-invasively in participants who decline blood collection, the ability to mail saliva collection kits to remote locations without requiring participants to visit a phlebotomy center, and stability of saliva samples for years at room temperature without the need for immediate DNA extraction, thereby allowing local storage and batched shipping. In addition, in patients who have been recently exposed to blood transfusions or hematopoietic stem cell transplantation from an allogeneic donor, or have significant leucopenia which can decrease the DNA yield from blood, saliva may be preferred to blood. The limitations of saliva samples are the lower DNA yield from saliva, microbial contamination if saliva is not properly collected, as well as the inability of younger children to provide saliva as per instructions.

To determine whether saliva-derived DNA can serve as an adequate substitute for blood-derived DNA, we compared WGS data from blood- and saliva-derived DNA from participants recruited into the Heart Centre Biobank Registry, a biorepository for childhood onset heart disease [4, 5]. Specifically, we compared the proportion of sequencing reads that map to non-human sources in blood versus saliva, the sequencing coverage between blood and saliva samples, and the concordance of single nucleotide variant (SNV) and copy number variant (CNV) calls between blood and saliva samples.

## Methods

### Study samples

Study participants were derived from the Heart Centre Biobank Registry, a multi-center biorepository that has been prospectively enrolling pediatric and adult patients with (or at risk for) heart disease since 2007 [4]. Five hundred thirty-one participants with blood derived DNA and 502 participants with saliva derived DNA undergoing WGS were analyzed. Paired blood and saliva samples from five unrelated individuals participating in the Biobank were directly compared for quality of WGS. Two (Sample Pairs 1 and 2) were female probands diagnosed with tetralogy of Fallot, a type of congenital heart disease, and three (Sample Pairs 3, 4 and 5) were male probands diagnosed with hypertrophic cardiomyopathy. Samples were collected during in-hospital visits. Participants were instructed to not eat, drink, or smoke for 30 min before saliva collection, and were asked to provide at least 2 ml of saliva into the collection tube with instructions to complete saliva collection within 30 min of opening the tube. The protocol was approved by the local Research Ethics Board at SickKids Hospital. Written informed consent was obtained from each participant and/or parent/legal guardian through the Heart Centre Biobank Registry (1000011232) and study protocols adhered to the Declaration of Helsinki.

### DNA quality

2–5 ml blood was collected in EDTA tubes and 2–4 ml saliva was collected using Oragene saliva kits after oral rinse. DNA was extracted from blood or saliva through Chemagic Star robotic system using a magnetic bead methodology at the SickKids Centre for Applied Genomics. Quality control checks were performed using Agarose Gel Electrophoresis and Nanodrop 2000 Spectrophotometer to verify DNA integrity. DNA quantification was measured using Qubit 3.0 Fluorometer to confirm DNA concentration. DNA samples were deemed to have met quality control (QC) thresholds if they had a single clear band on the agarose gel, a minimum DNA concentration of 20 ng/μl, and a 260/280 absorbance ratio greater than 1.3. A total of 1 μg of DNA (final volume 30 μl) at a minimum concentration of 20 ng/μl was used for WGS.

### Sequencing, read alignment, and variant calling

WGS was performed using Illumina HiSeq X to a target average coverage depth of 30x and a read length of 150 bp. The resulting reads were not filtered for minimum quality in order to avoid losing possible contaminant reads. Sequencing read alignment was done using Isaac Aligner to human genome build hg19. Short variant i.e. single-nucleotide variant (SNV) and small insertion-deletion (indel) calling was performed using Isaac Variant Caller with default parameters. To interpret variant pathogenicity, we implemented a variant prioritization pipeline based on the 2015 American College of Medical Genetics and Genomics (ACMG) variant interpretation criteria [6]. SNVs identified as pathogenic or likely pathogenic by the pipeline were manually confirmed for pathogenicity. Copy number variants (CNVs) were called using Control-FREEC [7] for Sample Pairs 1 and 2 or Canvas [8] for Sample Pairs 3, 4, and 5.

### Down-sampling

In order to account for possible bias resulting from differing numbers of reads between blood and saliva samples, each sample was randomly reduced to 730 million reads using samtools (version 1.4.1) [9]. All subsequent analyses on each pair of blood and saliva sequencing datasets were performed in complete datasets and in down-sampled datasets.

### Gene sets and regions

We compiled a list of 826 cardiovascular disorder-associated (CVD) genes which included genes represented in commercially available cardiovascular disease gene panels and previously published genes known to be associated with congenital heart disease, cardiomyopathy, and other cardiovascular diseases [10]. The genomic regions covered by the canonical transcripts for CVD genes, as well as for all genes, were obtained from the Consensus CDS (CCDS) database [11–13] (see Additional file 1: Table S1 for genomic positions of all CCDS transcripts and Additional file 1: Table S2 for genomic positions of CVD gene transcripts). For any CVD genes lacking transcripts in the CCDS database [11–13], we used the transcript start and end positions from Ensembl GRCh37 release 93 [14].

### Statistical analysis

#### Microbial contamination analysis

To find the extent of microbial contamination, we extracted the set of reads from each sample marked as unmapped to the hg19 reference genome using samtools (version 1.4.1) [9]. Then, using FastQ Screen (version 0.11.4) [15] running BWA (version 0.7.15) [16], we re-mapped the unmapped reads to the human reference genome hg19 and the microbial sequences from the Human Oral Microbiome Database (HOMD) [17] and compared the proportion of previously unmapped reads that remapped to the human genome versus the microbiome.

#### Coverage comparison

We analyzed genome-wide sequencing coverage for each WGS dataset using the genomecov command from the bedtools toolset (version 2.25.0) [18] on the aligned reads. We also used the coverage command from the bedtools toolset to find sequencing coverage within the regions covered by all canonical CCDS transcripts [11–13] and the canonical CCDS transcripts for the 854 CVD genes. All three analyses provided a coverage profile with the number of nucleotides covered at all sequencing depths for comparison between paired samples. We used the cumulative sum for each coverage profile to determine the number of positions covered at or greater than target depth and generated the curves for the cumulative coverage data using the statistical software R (version 3.5.1) [19]. We calculated the proportion of the genome covered at a minimum 20x coverage. Then, we used a paired t-test on the percentage of the whole genome, CCDS transcripts [11–13], and CVD gene transcripts covered at 20x or greater in order to find whether coverage in blood was significantly different from coverage in saliva.

#### Variant comparison

We used the view command from bcftools (version 1.4.1) [20] to filter the short variant calls in order to only retain SNVs, then the isec command to find the intersections of all SNVs called in each blood and saliva sample pair. We annotated SNVs unique to either blood or saliva with snpEff (version 4.3) [21] to computationally predict variant effects. We repeated this for SNVs falling within canonical CCDS transcripts [11–13] and within the canonical CCDS transcripts for the 854 CVD genes. Finally, we repeated each of the previous comparisons for rare SNVs, i.e. those that were absent or occurred at a minor allele frequency (MAF) of less than 1% in the Genome Aggregation Database (gnomAD) [22]. All SNVs, CNVs, and rare SNVs were compared for concordance between paired blood and saliva samples. Where clinical genetic test results had identified a pathogenic variant, we compared the detection rate of these known variants in blood versus saliva samples. In addition, we compared the proportion of variants that were concordant between paired blood and saliva and the types of variants, i.e. variants in exonic, intronic, intergenic, pseudogene regions, or causing gene fusions between blood and saliva. In order to find the concordance between CNVs called in each blood and saliva sample pair, we used the intersect command from bedtools (version 2.25.0) [18] in order to find which CNVs in each sample had > 50% overlap with at least one other CNV called in its counterpart.

## Results

Since the launch of the Heart Centre Biobank Registry in 2007, 7408 participants were recruited. Five hundred thirty-one blood and 502 saliva samples from recruited participants had DNA quality assessed prior to WGS for different research projects. Average DNA quality metrics for the blood and saliva samples are shown in Table 1. Overall, DNA from 46% saliva samples failed QC requirements for WGS and were not sequenced compared to only 6% QC failure for DNA from blood samples. The 5 paired blood and saliva samples passed quality metrics and showed comparable DNA quality between blood and saliva.

**Table 1:** DNA quality metrics for blood and saliva derived DNA

	Pooled samples		Paired samples	
	Blood DNA	Saliva DNA	Blood DNA	Saliva DNA
Number of Samples	531	502	5	5
Fluorometric DNA Concentration (ng/μL)	180	153	314	214
260/280 Absorbance Ratio	1.84	1.8	1.84	1.74
260/230 Absorbance Ratio	1.96	1.32	1.36	1.28
Samples Failing Mandatory Criteria	32 (6%)	231 (46%)	0 (0%)	0 (0%)
Samples Failing DNA Concentration cut-off (< 20 ng/μL)	0	0	0	0
Samples Failing 260/280 Absorbance Ratio (≤ 1.3)	0	0	0	0
Samples Failing Agarose Gel	32	231	0	0

### Microbial contamination analysis

**Fig. 1:**
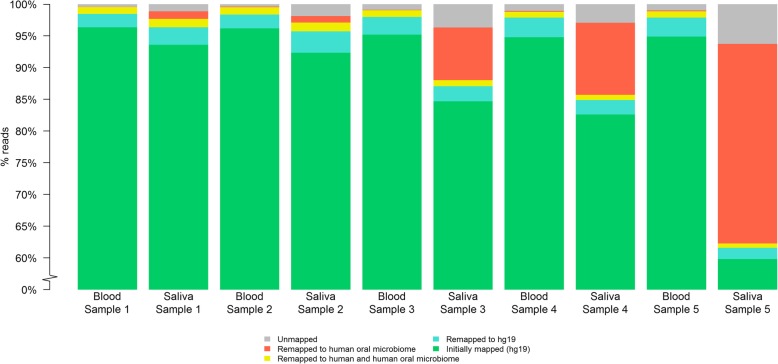
Proportion of reads mapping to the human reference genome hg19 and/or human oral microbiome for 5 blood and saliva pairs. Saliva sample 5 showed a high level of microbial contamination

An average of 95.5% of all WGS reads from blood samples and 82.6% of reads from saliva samples initially mapped to the hg19 human reference genome. Of the unmapped reads, 2.6% from blood and 2.5% from saliva samples re-mapped to the hg19 human reference using FastQ Screen [15] and BWA [16], 0.09 and 10.7% from blood and saliva respectively mapped to the human oral microbiome, and 1.05 and 1.03% of reads from blood and saliva respectively mapped to both hg19 and the human oral microbiome. Therefore, read mapping to the hg19 human reference genome was effective in excluding most of the reads from the human oral microbiome. The proportion of final mapped and unmapped reads in each sample are summarized in Fig. 1. Although a higher proportion of reads from saliva samples mapped exclusively to the human oral microbiome compared to blood samples, this difference was not statistically significant based on a paired t-test (*p* = 0.13). Of note, saliva sample 5 had the highest proportion of reads mapping to the human oral microbiome compared to the other samples. After excluding it, the final proportion of reads mapping to hg19 increased to 98.2% in blood and 93.8% in saliva.

### Coverage analysis

**Fig. 2:**
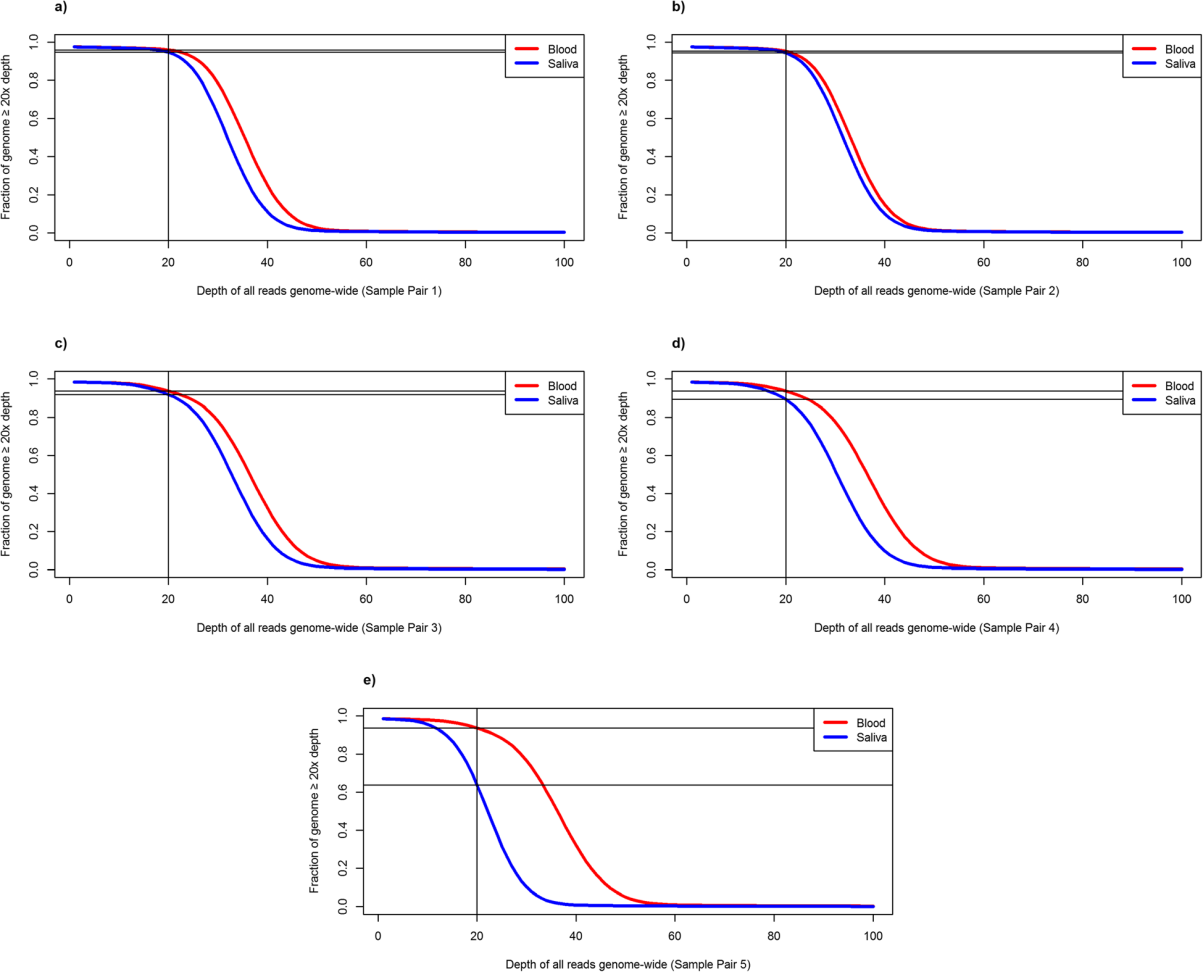
Average sequencing depth curves, displaying the cumulative proportion of the whole genome covered at a minimum of 20x sequencing depth for the entire set of reads in 5 paired blood and saliva samples (**a-e**). The vertical lines indicate 20x sequencing depth, while the horizontal lines indicate the fraction of the genome at 20x sequencing depth or greater

**Fig. 3:**
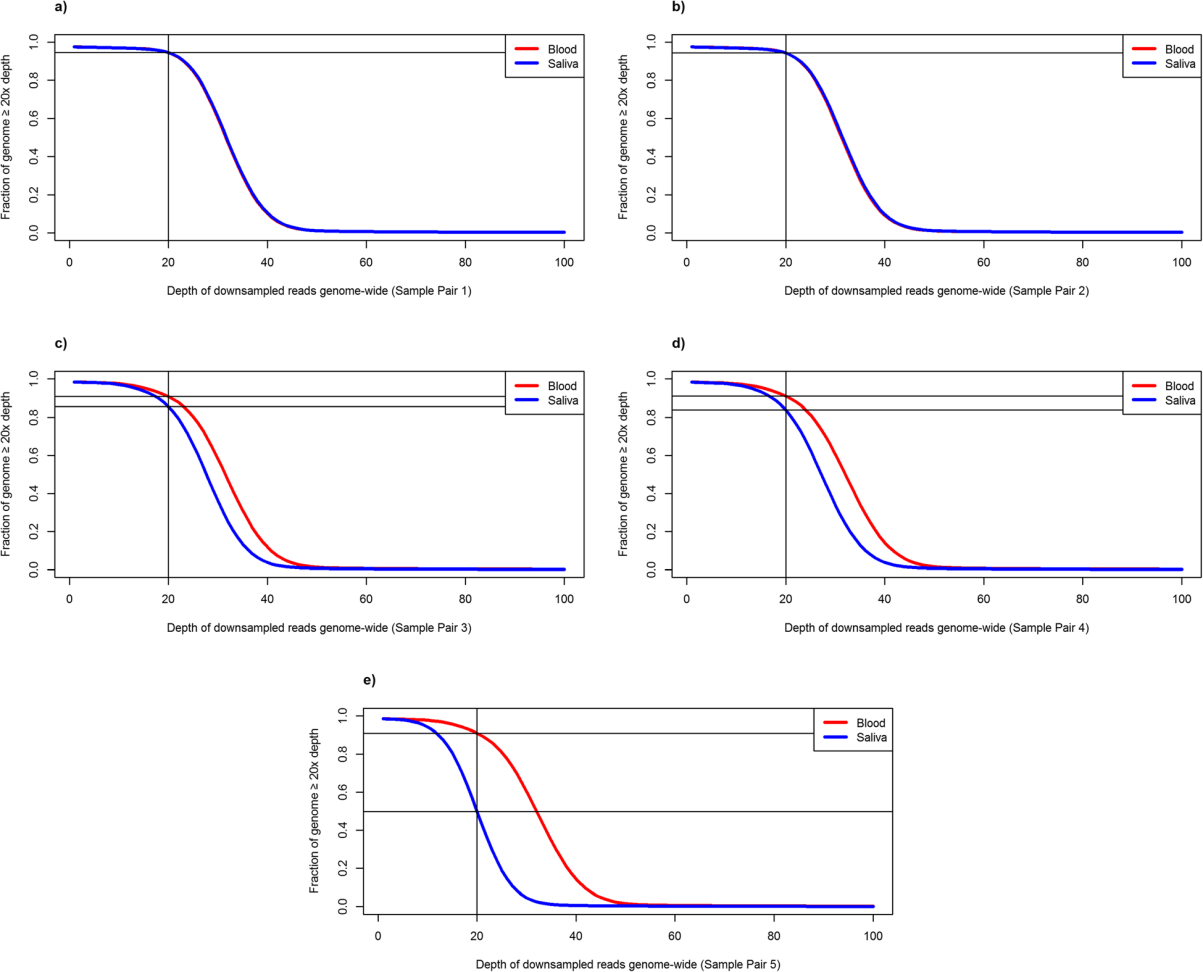
Average sequencing depth curves, displaying the cumulative proportion of the whole genome covered at a minimum of 20x sequencing depth of a randomly down-sampled set of 730 M reads in 5 paired blood and saliva samples (**a-e**). The vertical lines indicate 20x sequencing depth, while the horizontal lines indicate the fraction of the genome at 20x sequencing depth or greater

The reported mappable mean depth of coverage was more than 30x for all of the blood samples and 4 of the 5 saliva samples (Table 2). Saliva sample 5 had a lower mean depth of 22.6x. This was seen despite the DNA quality metrics being acceptable in saliva sample 5, which had a DNA concentration of 280 ng/μL, and a 260/280 absorbance ratio of 1.88. Excluding pair 5, the proportion of the genome with at least 20x coverage for all reads ranged from 93 to 96% for blood genomes and from 85 to 94% for saliva genomes (*p* = 0.07). The proportion of the genome with at least 20x coverage for 730 M randomly down-sampled reads ranged from 91 to 94% for blood genomes and from 84 to 94% for saliva genomes (*p* = 0.19). The proportion of CCDS transcript regions with mean coverage of at least 20x was 94.7% for blood and 92.5% for saliva (*p* = 0.12). The proportion of samples with mean coverage of at least 20x within the subset of transcripts for the 854 CVD genes was 96.9% for blood and 94.6% for saliva (*p* = 0.08). Therefore, saliva samples had overall adequate depth of coverage which was not significantly different from coverage in blood. The genome-wide cumulative coverage plots for the 5 paired samples, for all reads and the set of 730 M down-sampled reads, are displayed in Figs. 2 and 3. The cumulative coverage in Consensus CDS (CCDS) transcripts [11–13] for all reads and down-sampled reads, and in CVD gene regions for all reads and down-sampled reads, are shown in Additional files 2, 3, 4 and 5, respectively.

**Table 2:** Sequencing coverage in 5 sample pairs

	Sample Pair 1		Sample Pair 2		Sample Pair 3		Sample Pair 4		Sample Pair 5	
	Blood	Saliva	Blood	Saliva	Blood	Saliva	Blood	Saliva	Blood	Saliva
Mappable mean depth	36.5x	33.2x	34.2x	33.0x	37.5x	34.3x	37.0x	31.5x	36.8x	22.6x
Proportion of whole genome with at least 20x coverage for all reads	96.0%	94.6%	95.3%	94.3%	93.7%	85.6%	93.7%	89.3%	93.6%	63.8%
Proportion of whole genome with at least 20x coverage for 730 M down-sampled reads	94.5%	94.5%	94.3%	94.3%	90.8%	85.6%	91.1%	83.9%	91.0%	49.8%

### Variant yield between blood and saliva

#### All SNV concordance

**Fig. 4:**
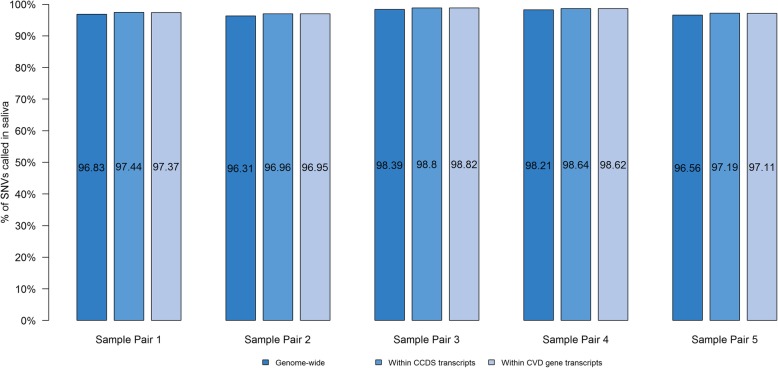
Proportion of single nucleotide variants in saliva genomes (*n* = 5). The bar graph shows average proportion of all SNVs called in blood genomes that were also detected in a paired saliva sample genome-wide, in CCDS transcripts, and in CVD gene transcripts

In light of a lower proportion of reads from saliva mapping to the human reference genome, we compared if variant yield was also lower in saliva than in blood. The average SNV yield in blood was 3.71 M and in saliva was 3.68 M (see Additional file 1: Table S3). In addition, the proportion of SNVs called in blood that were also detected in the paired saliva sample was > 95% for genome-wide calls, for SNVs in all CCDS transcripts [11–13], and for SNVs in CVD genes (see Fig. 4). Of the discordant SNVs, i.e. SNVs unique to either blood or saliva, only 0.6% were exonic; the remainder included 27% intronic, 65% intergenic, and 6.5% within a pseudogene (see Additional file 1: Table S3). There was no difference in the types of unique SNVs between blood and saliva samples.

#### Rare SNV concordance

**Fig. 5:**
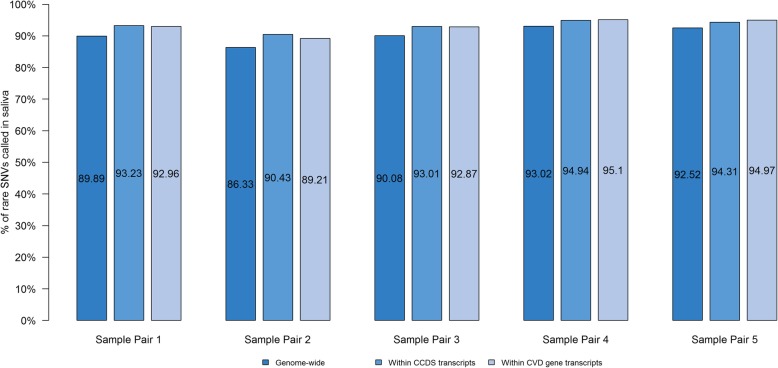
Proportion of rare single nucleotide variants (MAF < 1% in general population) in saliva genomes (*n* = 5). The bar graph shows average proportion of all rare SNVs called in blood genomes that were also detected in a paired saliva sample genome-wide, in CCDS transcripts, and in CVD gene transcripts

Proportion of rare SNVs in blood i.e. MAF < 1% that were also detected in saliva genomes was lower but over 85% as shown in Fig. 5. The average SNV concordance between blood and saliva genomes was 90.4 ± 3% genome-wide, 93.2 ± 2% in all CCDS transcripts [11–13], and 93 ± 2% in CVD genes. Further, rare pathogenic variants and variants of uncertain significance (VUS) identified on previous clinical genetic testing in the 3 hypertrophic cardiomyopathy samples were detected in both blood and saliva whole genome pairs from these 3 patients. These included a heterozygous pathogenic missense variant in *MYH7* (NM_000257.4:c.G1208A:p.R403Q) in Sample Pair 3, a heterozygous missense VUS in *MYBPC3* (NM_000256.3:c.T3548G:p.F1183C) in Sample Pair 4, and a heterozygous pathogenic missense variant in *MYH7* (NM_000257.4:c.C2722G:p.L908 V) in Sample Pair 5. The comparisons for all SNVs over the entire genome, in CCDS transcripts [11–13], and CVD gene transcripts as well as rare SNVs in the aforementioned regions, are described in Additional file 1: Tables S4-S9.

#### CNV concordance

**Fig. 6:**
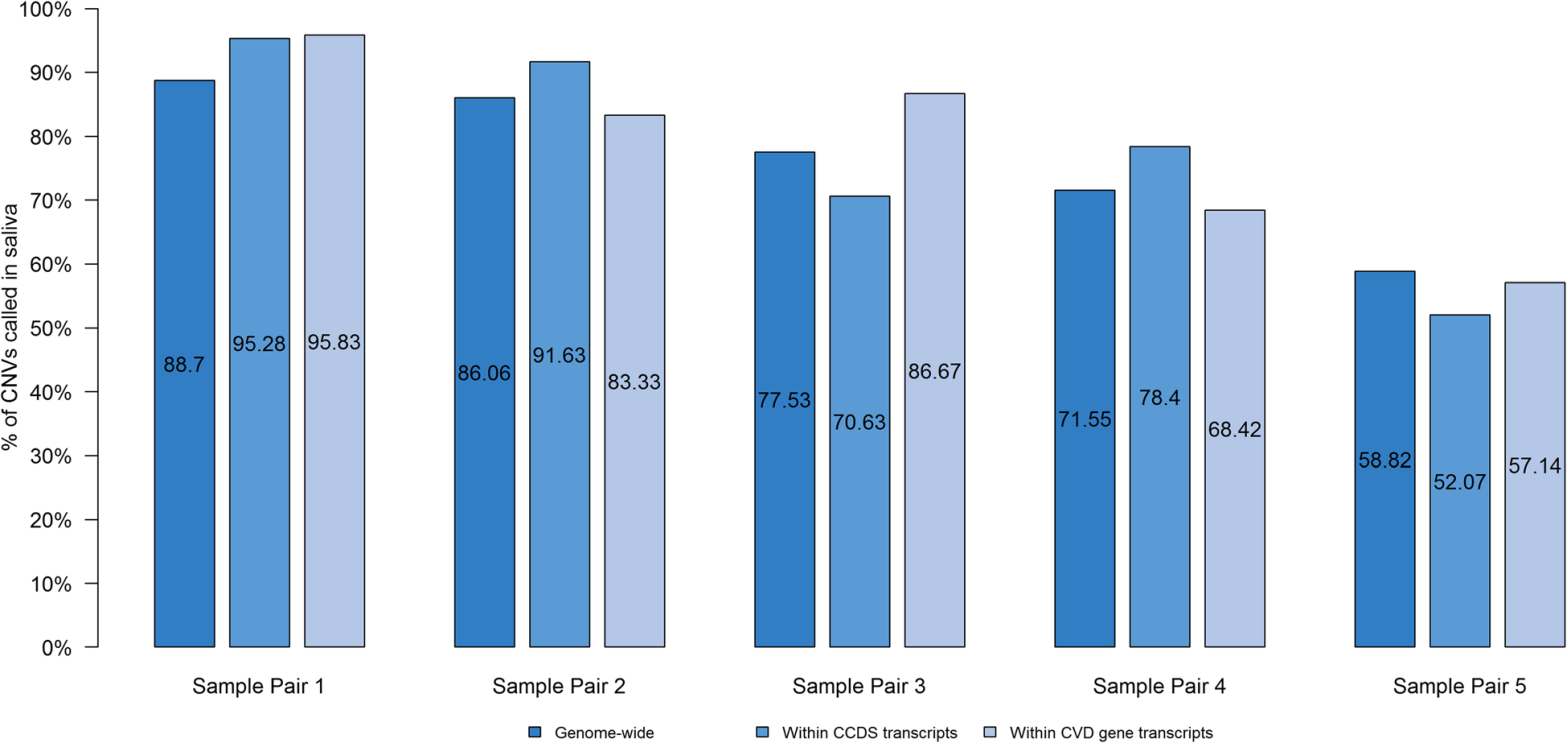
Proportion of copy number variants in saliva genomes (*n* = 5). The bar graph shows average proportion of all CNVs called in blood genomes that were also detected in a paired saliva sample genome-wide, in CCDS transcripts, and in CVD gene transcripts

Overall there was considerable variability in CNV calling rates across both blood and saliva samples. The average number of CNV calls were 14% lower in saliva compared to blood. The average proportion of CNVs in blood that were also detected in saliva genomes was lower at 76% genome-wide, 77% in all CCDS transcripts [11–13], and 78% in CVD genes (see Fig. 6). The comparison for all CNVs in each sample across the entire genome, in CCDS transcripts [11–13], and CVD gene transcripts including shared and unique CNVs are described in Additional file 1: Table S10. CNV detection was lowest in Sample 5 (< 60%) compared to the other 4 sample pairs.

## Discussion

The advances in genomics research has increased the need for collaboration and sample sharing amongst researchers. Population biorepositories often acquire samples from participants remotely. Saliva can be stored for long periods of time and shipped at room temperature which makes sample preservation during shipping easier compared to blood. However, there has been little evidence regarding the suitability of saliva samples for WGS. The study by Wall et al. [3] found “no difference in the sequencing quality or error rate of blood and saliva samples”, but they did not perform a systematic comparison of the coverage, microbial contamination, or variant concordance in paired blood and saliva samples. Our results showed that a higher proportion of saliva samples (almost 50%) may not meet the stringent QC criteria required for WGS. However for samples that do meet stringent QC criteria, the quality of WGS SNV data from saliva samples is comparable to blood samples in majority of the samples (80% in our study). Our study showed that saliva genomes derived from high quality DNA had good coverage, low microbial contamination and a 90–95% concordance for sequence variant calls, but lower than 80% for CNVs.

When comparing for specific differences between paired samples, we found higher microbial contamination in saliva-derived DNA, as shown by a higher proportion of short-read mapping to the sequences from the Human Oral Microbiome Database (HOMD) [17], although this was not statistically different. However, since these reads typically do not map to the human reference genome and remain unmapped during read alignment, they are not included in downstream analyses and therefore are unlikely to have a major effect on variant calling. There remains a concern that reads mapping to the microbiome in saliva samples compete with the mapping of reads to the human genome thereby reducing the resolution of human relevant sequence data. This can be addressed by increasing the target coverage depth with higher resolution sequencing. Our findings suggest that this may not be routinely required for all saliva samples since the WGS quality was comparable to blood in the majority of saliva samples. In particular, rare clinically relevant variants identified on clinical genetic testing were detectable in saliva and blood genomes from the same patients.

We further found that in 4 of the 5 pairs (80%), the proportion of the genome covered at 20x or greater was similar between blood and saliva genomes, with only 2% higher coverage in blood versus saliva at 20x both across the genome as well as within all CCDS transcripts [11–13] and CCDS transcripts for 854 CVD genes. Of note, Saliva Sample 5 had a higher proportion of reads that mapped exclusively to the human oral microbiome which caused the average read coverage to drop to 22.6x, which is below the average target depth of 30x. This may reflect improper saliva collection technique with more oral microbiome contamination at the time of saliva collection, although there was no evidence of this since the agarose gel results, DNA concentration, and 260/280 absorbance ratios were comparable between the blood and saliva sample. In a similar series of comparisons involving whole exome sequencing, Zhu et al. [23] found that sequences from blood had a 3.3% higher proportion with minimum 20x coverage in blood compared to saliva but this was not significantly different. With randomly down-sampling each genomic sequence in order to ensure an equal number of reads between paired samples, there remained a non-significant difference in coverage between blood and saliva genomes, and this difference was lower than the difference reported in exome comparisons by Zhu et al. [23].

Reassuringly, despite the differences in coverage and microbial contamination in one of the 5 saliva samples, WGS from saliva samples was able to detect 95% of all SNVs detected in a paired blood sample for SNVs genome-wide, within CCDS transcripts [11–13], and within CVD gene transcripts. When comparing rare SNVs (MAF < 1%), the proportion of shared variants seen in saliva dropped to 90% for all rare SNVs, and 93% for SNVs in CCDS transcripts [11–13] and in CVD genes. This limitation needs to be kept in mind when using saliva samples for WGS. Nevertheless, it was reassuring that rare causal SNVs and VUS identified on clinical testing could still be detected by WGS in both blood and saliva samples. There were no systematic differences between functional categories of SNV calls between blood and saliva genomes.

CNV yield was more variable across blood and saliva samples with lower proportion of shared CNVs between blood and saliva – 76% for all CNVs, 77% for CNVs in CCDS transcripts [11–13], and 78% for CNVs in CVD genes. The lower CNV yield in saliva samples may be the result of the reduced coverage in the saliva sample since both Control-FREEC and Canvas utilize read depth in order to call CNVs [7, 8]. As a result, a reduction in read depth may have a larger effect on CNVs calling in a given dataset. On the other hand, the lack of a best practices pipeline for CNV calling [24] may also be a contributing factor to the observed inconsistency in CNV calls.

## Conclusions

In summary, there was a higher proportion of saliva-derived DNA that failed QC for WGS compared to blood-derived DNA. However, saliva DNA that met stringent QC metrics generated good quality WGS data with comparable detection of common and rare SNVs despite evidence of oral microbiome sequence in some saliva samples. Unlike SNV calls, CNV yield in saliva was lower than that in blood. Given the greater variability in DNA quality from saliva samples, it is critical to ensure proper technique for saliva collection, apply stringent QC criteria to DNA quality, and verify that target sequencing depth has been met and that at least 90% of reads map to the human genome in WGS data from saliva samples as a minimum threshold for use in downstream analysis. Use of microbiome kit and/or higher depth of sequencing may help WGS yield in samples not meeting QC metrics; however this was not explored in our study. Our findings suggest that while blood remains the preferred source of DNA for WGS, DNA from saliva samples that meets quality filters can serve as an acceptable alternative for WGS primarily for SNV calls, when blood samples are not available or not suitable.

## Supplementary information

**Additional file 1:** **Table S1.** Coordinates of all Consensus Coding Sequence (CCDS) transcripts in the hg19 genome build. **Table S2.** Coordinates of all cardiovascular disease (CVD) gene transcripts in the hg19 genome build. **Table S3.** Concordance of SNVs and CNVs between saliva and blood whole genomes. **Table S4.** Comparison of SNVs genome-wide in each paired blood and saliva genome. **Table S5.** Comparison of SNVs within CCDS transcripts in each paired blood and saliva genome. **Table S6.** Comparison of SNVs within CVD gene transcripts in each paired blood and saliva genome. **Table S7.** Comparison of rare SNVs genome-wide in each paired blood and saliva genome. **Table S8.** Comparison of rare SNVs within CCDS transcripts in each paired blood and saliva genome. **Table S9.** Comparison of rare SNVs within CVD gene transcripts in each paired blood and saliva genome. **Table S10.** Comparison of all CNVs genome-wide, within CCDS transcripts, and within CVD gene transcripts.

**Additional file 2:** Cumulative coverage for all reads in CCDS transcripts in paired blood and saliva genomes.

**Additional file 3:** Cumulative coverage for down-sampled reads in CCDS transcripts in paired blood and saliva genomes.

**Additional file 4:** Cumulative coverage for all reads in cardiovascular disorder (CVD) gene transcripts in paired blood and saliva genomes.

**Additional file 5: **Cumulative coverage for down-sampled reads in CVD transcripts in paired blood and saliva genomes**.**

## Data Availability

The datasets generated and analyzed during the current study are available in the European Genome-Phenome Archive (Accession number EGAS00001004115).

## Abbreviations

ACMG: American College of Medical Genetics and Genomics
CCDS: Consensus CDS
CDS: Coding sequence
CNV: Copy number variant
CVD: Cardiovascular disorder
DNA: Deoxyribonucleic acid
HOMD: Human Oral Microbiome Database
MAF: Minor allele frequency
QC: Quality control
SNV: Single-nucleotide variant
VUS: Variant of unknown/uncertain significance
WES: Whole exome sequencing
WGS: Whole genome sequencing

## References

[1] Bahlo M, Stankovich J, Danoy P, Hickey PF, Taylor BV, Browning SR (2010). Saliva-derived DNA performs well in large-scale, high-density single-nucleotide polymorphism microarray studies. Cancer Epidemiol Biomark Prev.

[2] Kidd JM, Sharpton TJ, Bobo D, Norman PJ, Martin AR, Carpenter ML (2014). Exome capture from saliva produces high quality genomic and metagenomic data. BMC Genomics.

[3] Wall JD, Tang LF, Zerbe B, Kvale MN, Kwok PY, Schaefer C (2014). Estimating genotype error rates from high-coverage next-generation sequence data. Genome Res.

[4] Fung A, Manlhiot C, Naik S, Rosenberg H, Smythe J, Lougheed J (2013). Impact of prenatal risk factors on congenital heart disease in the current era. J Am Heart Assoc.

[5] Papaz T, Safi M, Manickaraj AK, Ogaki C, Breaton Kyryliuk J, Burrill L (2012). Factors influencing participation in a population-based biorepository for childhood heart disease. Pediatrics..

[6] Richards S, Aziz N, Bale S, Bick D, Das S, Gastier-Foster J (2015). Standards and guidelines for the interpretation of sequence variants: a joint consensus recommendation of the American College of Medical Genetics and Genomics and the Association for Molecular Pathology. Genet Med.

[7] Boeva V, Popova T, Bleakley K, Chiche P, Cappo J, Schleiermacher G (2012). Control-FREEC: a tool for assessing copy number and allelic content using next-generation sequencing data. Bioinformatics..

[8] Roller E, Ivakhno S, Lee S, Royce T, Tanner S (2016). Canvas: versatile and scalable detection of copy number variants. Bioinformatics..

[9] Li H, Handsaker B, Wysoker A, Fennell T, Ruan J, Homer N (2009). The sequence alignment/map format and SAMtools. Bioinformatics..

[10] Andersen TA, Troelsen Kde L, Larsen LA (2014). Of mice and men: molecular genetics of congenital heart disease. Cell Mol Life Sci.

[11] Pruitt KD, Harrow J, Harte RA, Wallin C, Diekhans M, Maglott DR (2009). The consensus coding sequence (CCDS) project: identifying a common protein-coding gene set for the human and mouse genomes. Genome Res.

[12] Harte RA, Farrell CM, Loveland JE, Suner MM, Wilming L, Aken B (2012). Tracking and coordinating an international curation effort for the CCDS Project. Database (Oxford).

[13] Farrell CM, O'Leary NA, Harte RA, Loveland JE, Wilming LG, Wallin C (2014). Current status and new features of the consensus coding sequence database. Nucleic Acids Res.

[14] Zerbino DR, Achuthan P, Akanni W, Amode MR, Barrell D, Bhai J (2018). Ensembl 2018. Nucleic Acids Res.

[15] Wingett SW, Andrews S (2018). FastQ Screen: A tool for multi-genome mapping and quality control. F1000Res.

[16] Li H, Durbin R (2009). Fast and accurate short read alignment with burrows-wheeler transform. Bioinformatics..

[17] Chen T, Yu WH, Izard J, Baranova OV, Lakshmanan A, Dewhirst FE (2010). The Human Oral Microbiome Database: a web accessible resource for investigating oral microbe taxonomic and genomic information. Database (Oxford).

[18] Quinlan AR, Hall IM (2010). BEDTools: a flexible suite of utilities for comparing genomic features. Bioinformatics..

[19] R Core Team (2018). R: a language and environment for statistical computing.

[20] Li H, Handsaker B, Danecek P, McCarthy S, Marshall J. bcftools. Version 1.4.1 [software]. 2017 May 8 [cited 2018 November 7]. Available from: https://www.nlm.nih.gov/bsd/uniform_requirements.html.

[21] Cingolani P, Platts A, Wang le L, Coon M, Nguyen T, Wang L (2012). A program for annotating and predicting the effects of single nucleotide polymorphisms, SnpEff: SNPs in the genome of Drosophila melanogaster strain w1118; iso-2; iso-3. Fly (Austin).

[22] Lek M, Karczewski KJ, Minikel EV, Samocha KE, Banks E, Fennell T (2016). Analysis of protein-coding genetic variation in 60,706 humans. Nature..

[23] Zhu Q, Hu Q, Shepherd L, Wang J, Wei L, Morrison CD (2015). The impact of DNA input amount and DNA source on the performance of whole-exome sequencing in cancer epidemiology. Cancer Epidemiol Biomark Prev.

[24] Trost B, Walker S, Wang Z, Thiruvahindrapuram B, MacDonald JR, Sung WWL (2018). A comprehensive workflow for read depth-based identification of copy-number variation from whole-genome sequence data. Am J Hum Genet.

