# Supplementary figures and images for: Quality of whole genome sequencing from blood versus saliva derived DNA in cardiac patients
Source: BMC Med Genomics. 2020 Jan 29;13:11. doi: 10.1186/s12920-020-0664-7 (PMC6988365; doi:10.1186/s12920-020-0664-7)

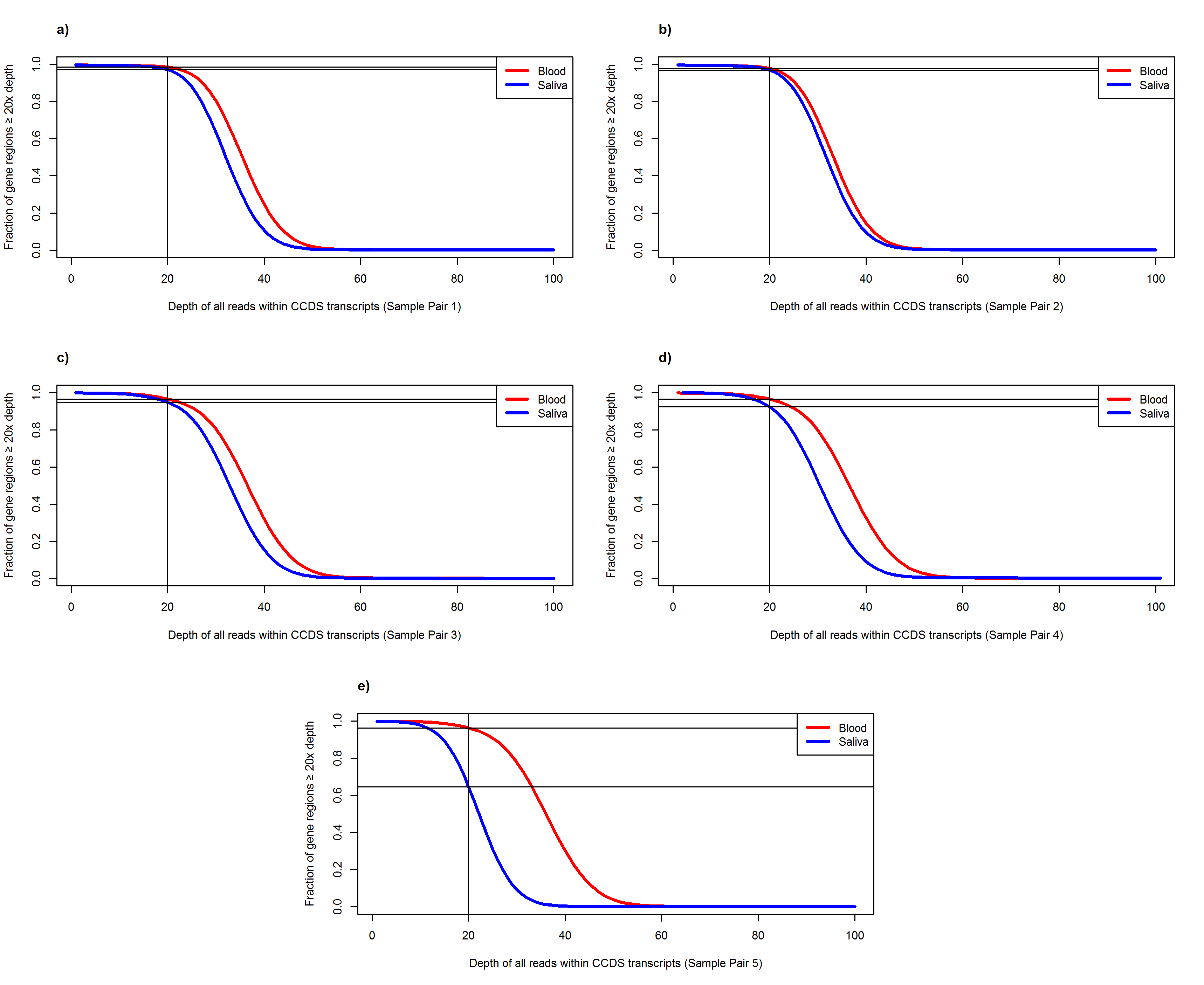

Supplement: Supplementary file 2 — Additional file 2: Cumulative coverage for all reads in CCDS transcripts in paired blood and saliva genomes. [file 12920_2020_664_MOESM2_ESM.tiff]

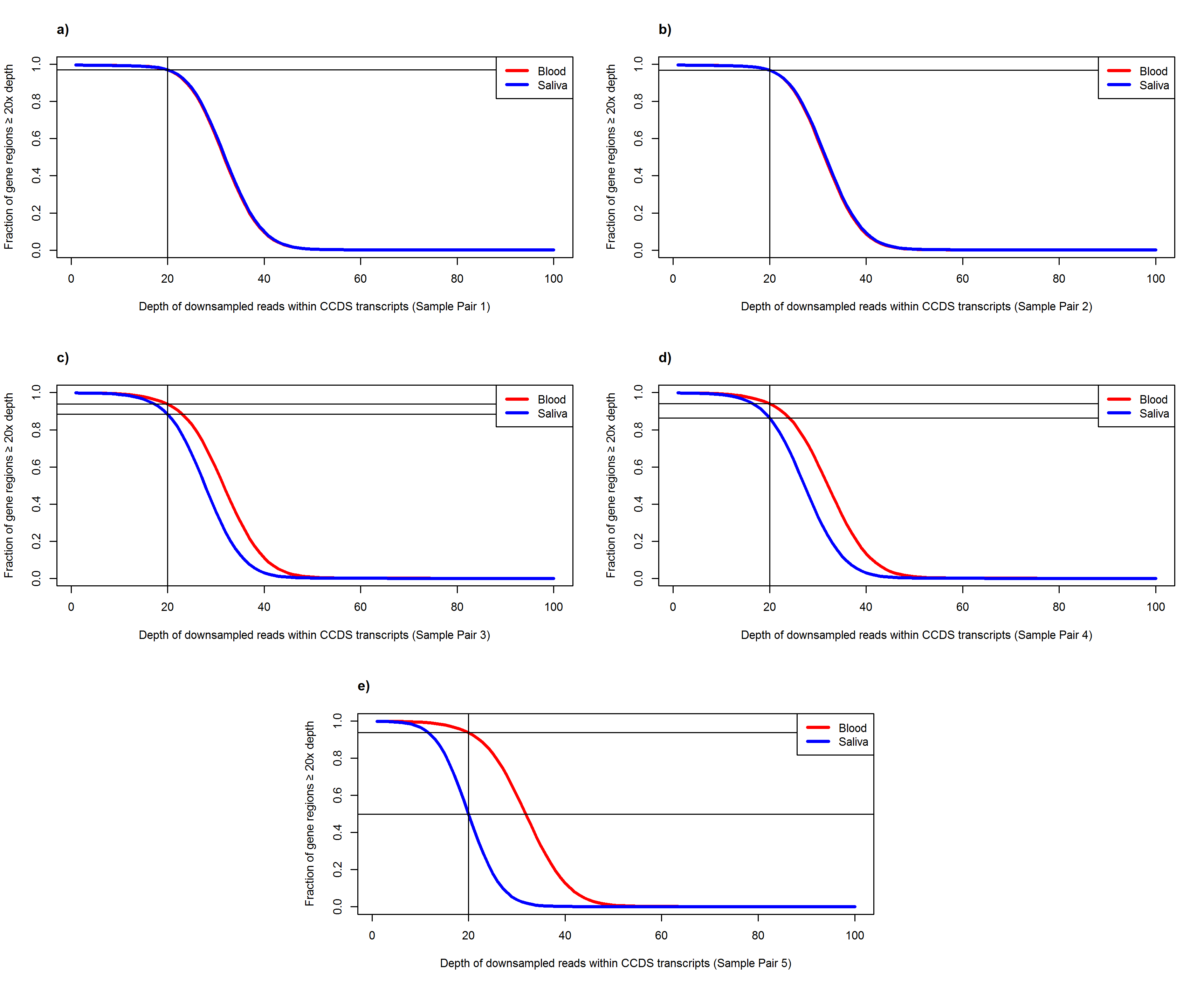

Supplement: Supplementary file 3 — Additional file 3: Cumulative coverage for down-sampled reads in CCDS transcripts in paired blood and saliva genomes. [file 12920_2020_664_MOESM3_ESM.tiff]

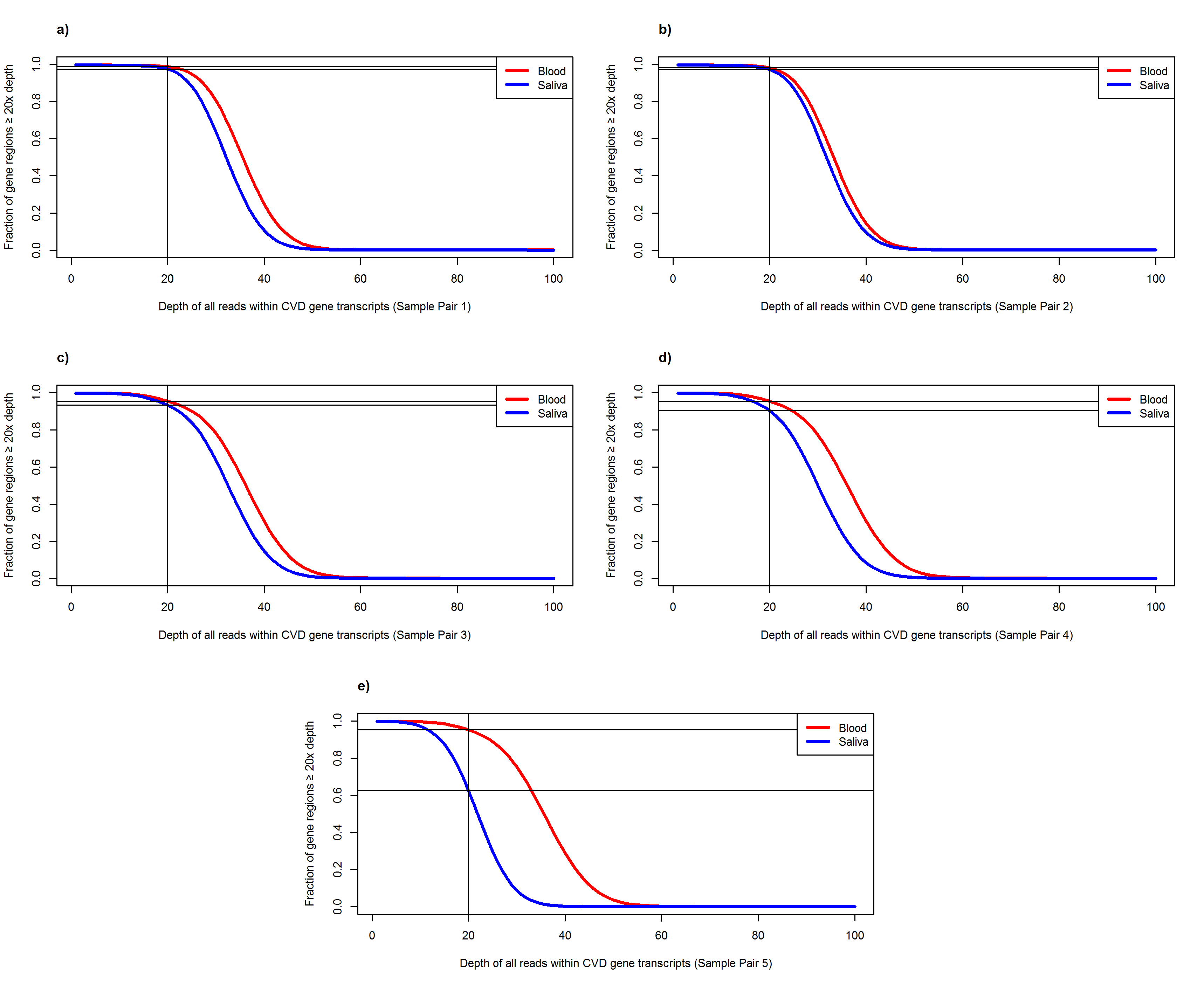

Supplement: Supplementary file 4 — Additional file 4: Cumulative coverage for all reads in cardiovascular disorder (CVD) gene transcripts in paired blood and saliva genomes. [file 12920_2020_664_MOESM4_ESM.tiff]

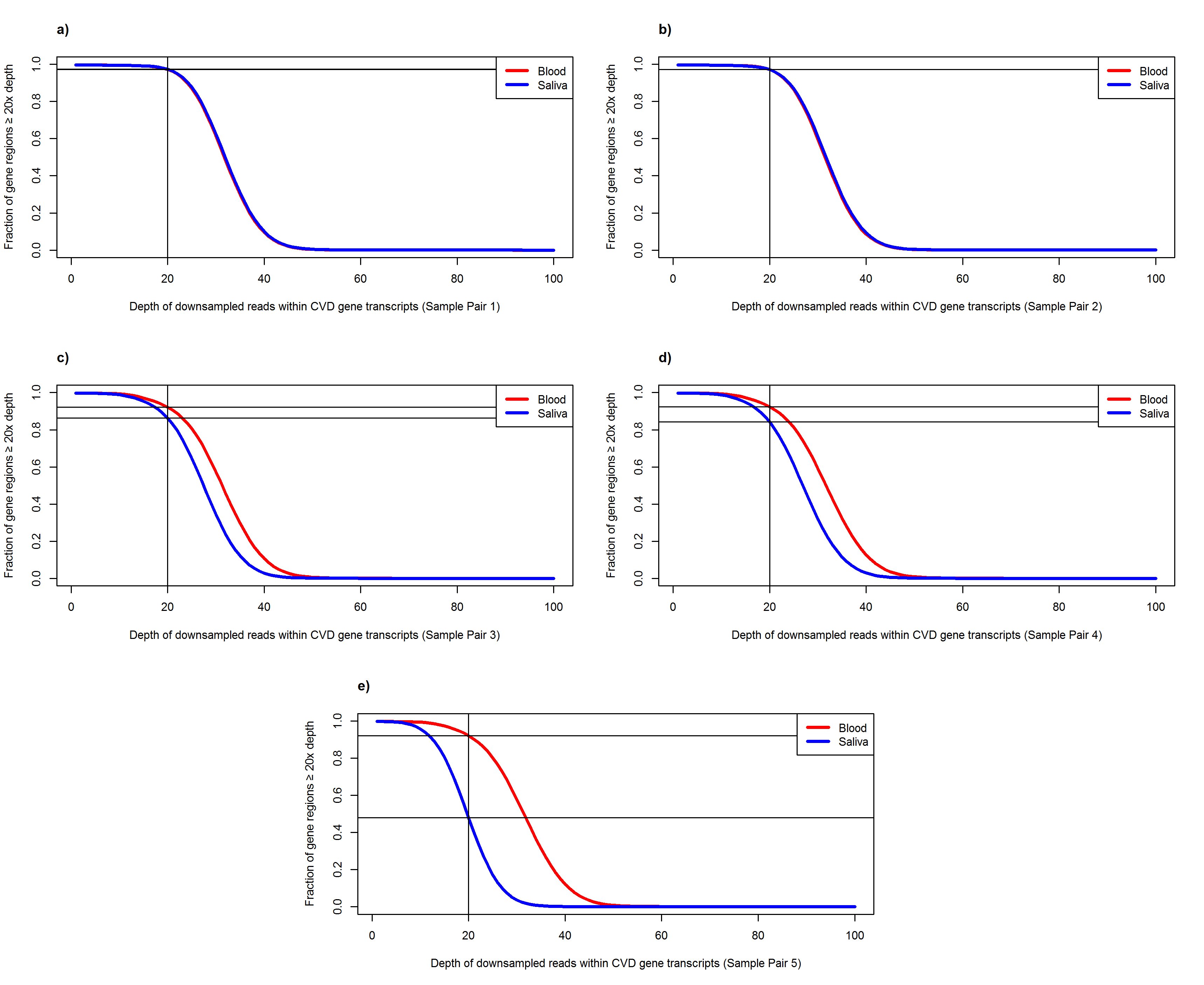

Supplement: Supplementary file 5 — Additional file 5: Cumulative coverage for down-sampled reads in CVD transcripts in paired blood and saliva genomes. [file 12920_2020_664_MOESM5_ESM.tiff]
